# Profiles of Emotion Regulation and Post-Traumatic Stress Severity among Female Victims of Intimate Partner Violence

**DOI:** 10.3390/ijerph18136865

**Published:** 2021-06-26

**Authors:** Marina Muñoz-Rivas, Ana Bellot, Ignacio Montorio, Rosa Ronzón-Tirado, Natalia Redondo

**Affiliations:** Faculty of Psychology, Autonomous University of Madrid, 28021 Madrid, Spain; ana.bellot@uam.es (A.B.); ignacio.montorio@uam.es (I.M.); rc.ronzon@uam.es (R.R.-T.); natalia.redondor@uam.es (N.R.)

**Keywords:** intimate partner violence, post-traumatic stress, emotional regulation, revictimization

## Abstract

Emotional dysregulation is a construct that has drawn substantial attention as a transdiagnostic contributing factor to the loss of health. Intimate partner violence (IPV) is a term used to describe physical, psychological, or sexual assault of a spouse or sexual partner. The aim of the study was to determine the variability of emotional dysregulation among women with different types of IPV revictimization and post-traumatic stress. The cross-sectional survey included 120 women attended by the Integrated Monitoring System of Gender Violence of Madrid, Spain, due to a gender violence complaint. The presence of post-traumatic stress disorder (DSM 5 criteria), emotional dysregulation (Emotional Processing Scale (EPS)), childhood trauma, and type of revictimization were evaluated. Cluster analysis found three profiles of emotional regulation: Emotionally Regulated, Avoidance/Non-Impoverished, and Emotional Overwhelm. The results showed that the Emotional Overwhelm group was characterized by a general dysregulation of emotional experiences and a greater intensity of post-traumatic stress symptoms. In addition, women who have suffered several episodes of IPV by different partners showed a differential pattern of emotional regulation than the rest of the victims that entailed greater psychopathology. Findings confirm that emotional dysregulation is a critical pathway to the decrease of health among IPV victims.

## 1. Introduction

### 1.1. Intimate Partner Violence

Intimate partner violence (IPV) against women has its roots in gender inequality and is currently considered as a violation of women’s human rights [1]. The World Health Organization considers IPV to be any behavior of an intimate partner or ex-partner that causes physical, sexual, or psychological harm to women (e.g., physical assault, sexual coercion, psychological abuse, and controlling behaviors), and estimates that one in three women in the world are affected by IPV during their lives [2].

IPV is a problem of global concern that negatively affects people beyond the immediate harm they suffer, with serious consequences on their physical health, the most frequent being chronic pain, hypertension, substance abuse, and diabetes [3,4,5,6], and their mental health, including sleep disturbances, depression and anxiety, post-traumatic stress, and suicide attempts [3,7,8,9,10]. In addition, it has been shown that the frequency and intensity with which victims suffer IPV directly affect the severity of such consequences. In addition, violence perpetrated by an intimate partner can cause more psychological harm than violence perpetrated by a stranger since it is linked to an individual the victim trusts, and it can hardly be justified as a random anonymous attack [11].

### 1.2. Post-Traumatic Stress Disorder (PTSD) and IPV

One focus of interest in the study of victims of IPV has been post-traumatic stress disorder (PTSD), both because of its high prevalence and the significant functional deterioration it entails, as well as the tendency for it to become chronic in the absence of treatment [12]. Prevalence rates of post-traumatic stress disorder in women victims of IPV range from 24% to 84% [13,14,15], with more serious clinical symptoms among the victims with more severe and frequent types of victimization [16]. In addition, when comparing IPV victims to women without a history of IPV, the former are three times more likely to have symptoms of post-traumatic stress, placing them in a more vulnerable situation [17]. Furthermore, when comparing IPV victims to victims of other trauma experiences, the former display more symptoms of post-traumatic stress that also do not disappear after the relationship is over [18].

### 1.3. Emotional Regulation

Often, people with diagnosed PTSD report different types of emotional problems. Firstly, in recalling the traumatic incident, they report negative emotional reactions such as sadness, shame, guilt, or anger—emotions that have, in fact, been incorporated as diagnostic criteria [19]. Secondly, research lends support to the association between PTSD and constructs of emotional value, such as: suppression (inhibition of expression of emotional responses [20]), experiential avoidance (attempt to suppress unwanted internal experiences such as emotions, thoughts, memories, and bodily sensations [21]), alexithymia (difficulty identifying what they feel [22]), or dissociation (mental process of disconnection from thoughts, feelings, memories, or sense of identity [23]). Thirdly, emotional numbness defined both by disaffection for emotion-provoking circumstances and by a lack of emotional reactivity has proved to be a relevant predictor of the endurance of post-traumatic symptoms [24].

Emotional regulation (ER) is a term used to describe the processes through which people influence which emotions they have, when they have them, how they experience them, and how they express them [25]. Hence, the regulation of emotions is multifaceted and characterized by the awareness, identification, and expression of emotional experiences, the ability to pay attention to contextual signals, the ability to control impulses, and the use of strategies to regulate emotions in the face of triggering emotional situations [26]. Deficits in emotional regulatory skills are related to greater negative affect, a decrease in positive affect, and the ineffectiveness of managing one’s emotions [27]. So far, research on traumatic events has focused on studying the role of strategies for regulating emotions in the development of trauma-related psychopathology. Thus, a recent meta-analysis found that PTSD, regardless of the type of traumatic incident that generates it, is characterized by a general dysregulation of emotions, and that this generalized dysregulation is more relevant in explaining the symptoms than any of the specific regulatory strategies used individually [28]. In addition, this same study points out that rumination, suppression, and experiential avoidance are the specific strategies most strongly associated with PTSD. Only a limited number of studies have examined the direct relationship between difficulties regulating emotions among women exposed to incidents of IPV and a PTSD diagnosis. The scant empirical evidence shows that women with IPV experiences have greater difficulty accepting their emotions and excessively employ emotional avoidance coping behaviors, such as dissociation, substance abuse, and/or self-harm, to reduce negative affect [29]. More recently, it has been observed that there is a general difficulty in regulating emotions among adult women with PTSD symptoms and victims of IPV that might stem from childhood abuse, since this is conducive to emotional dysregulation during development and could endure into adulthood [30].

### 1.4. Revictimization and Emotional Regulation

The reported relationship between IPV and the presence of PTSD becomes more relevant if we consider that between 22% and 46% of victims suffer from continuous traumatic experiences of abuse and medical treatment, making them up to 4–5 times more likely to have a new episode of psychological and/or physical victimization at the hands of the same or different partners in the 6 months following the previous abuse event [31,32]. In Spain, 18% of the cases of gender-based violence investigated by the police between 2007 and 2015 were victims with a previous history of victimization, and, of that percentage, 29.62% had been abused by several of their partners [31,32,33]. In this context, it cannot be forgotten that exposure to multiple traumatic experiences seriously affects a person’s ability to recover from subsequent traumatic events, and this fact is also evident in revictimized women, who present greater difficulties in identifying and managing their emotional states, with emotional numbness being highly characteristic of such cases [34,35,36]. In addition, it has been suggested that not only is dysregulation of emotion a sensitive indicator of IPV revictimization, but also that the negative impact of victimization experiences on adult emotional regulatory capabilities can be cumulative [37,38].

### 1.5. Current Study

Despite all the advances made in this field, research linking emotional regulation to PTSD has not consistently demonstrated how both factors work together to contribute to psychopathology, nor does it evaluate the joint emotional regulatory efforts of people who have experienced traumatic incidents. Currently, fewer than a dozen empirical studies report findings on three or more emotional regulation strategies together, and only a small part of them report findings in relation to post-traumatic experiences [39]. In addition, in the case of IPV incidents, the number of studies examining the direct relationship between difficulties regulating emotions among victims and PTSD is even more limited [30]. Given that people often use multiple strategies of emotional regulation simultaneously [40], the scarcity of studies investigating ER leaves a critical gap in the literature. Seligowski et al. [28], after reviewing research on emotional regulation and PTSD by means of a meta-analysis, conclude that clinical sample research should be a priority to explore the extent to which results are generalized, as many of the studies have been conducted with samples that are difficult to generalize (e.g., students). In addition, they recommend that such studies be conducted in relation to specific traumatic events. Furthermore, many of the studies with IPV victims have focused exclusively on victimization experiences without considering possible associations between different types of victimization and emotional regulation. For all of these reasons, the objective of this study was to examine the intensity of post-traumatic stress symptoms in a sample of women victims of intimate partner violence, as well as to evaluate five strategies of emotional regulation across the victims in order to identify specific profiles of emotional regulation related to IPV and the post-traumatic clinical symptoms observed. The strategy to identify emotional regulation profiles with different effects on post-traumatic stress has already been established [39,41,42], although not specifically in IPV events. The existence of at least three profiles of emotional regulation is hypothesized among women victims of IPV associated differentially with symptoms of post-traumatic stress and with the different types of IPV victims.

## 2. Materials and Method

### 2.1. Participants

The study sample was made up of 120 victims of intimate partner violence registered in the Integrated Monitoring System of Gender Violence Cases (VioGen) managed by Spain’s Ministry of the Interior in the municipality of Madrid.

The average age of the women was 38.5 years, 68.5% had Spanish or dual nationality including Spanish, 59.5% of the participants had a high school or university education, 26.4% had vocational training, 13.2% primary education, and less than 1% had no education, and 76.9% of the sample had children.

Of all participants, 51 women were victims of a single episode of gender violence (victims with a single report—VSR): women victims included in VioGen since 2014 who, after a single complaint, have not filed any further ones. Forty-nine women were victims of several episodes of gender-based violence perpetrated by the same partner (victims with several reports by the same aggressor, VSRSA): women victims included in VioGen since 2014 who have filed several complaints about the same aggressor, and 20 were victims of several episodes of gender violence perpetrated by multiple partners (victims with several reports by different aggressors, VSRDA: women victims included in VioGen since 2014 who have filed several complaints about different aggressors.

### 2.2. Procedure

This study was developed in collaboration with Spain’s Ministry of the Interior, responsible for the VioGen system, which records any complaints made by a women of gender-based violence at the national level. For each complaint, the victim’s data are registered by the police department which maintains the legal custody of victims’ data, history of victimization, and the corresponding legal and judicial measurements. After requesting permission to the Spain’s Ministry of Interior, one of the members of the research team was authorized to access to VioGen data under the supervision of the police department to select potential participants for the study. Thus, throughout the whole study, only the police officers and the authorized research team member had access to the VioGen data. The selection of the potential participants followed three inclusion criteria: (1) women with an active case in the VioGen system due to a gender violence complaint, (2) women with a judicial ruling of police protection measures, and (3) women had to be 18 years of age or older. After the selection, police officers were asked by the research team to inform the potential participants about the possibility of taking part in the study. Women who agreed to participate and allowed the police department to share their telephone number and alias with the research team were contacted via telephone by a member of the research team to obtain voluntary consent for their participation. After the participation agreement, an interview was scheduled according to the women’s availability. The interviews lasted approximately two hours and were conducted at the participants’ preferred location. Having signed the informed consent, the interview was conducted, structured, and guided by a standard evaluation protocol. The procedure was approved by the Research Ethics Committee of the Universidad Autónoma de Madrid (IEC-941720).

### 2.3. Instruments

Questionnaire on the psychosocial data of the victims: an ad-hoc questionnaire was used to collect information on: age, nationality, number of years living in Spain, marital status, level of education, work, and time elapsed since the complaint was filed.

Emotional Processing Scale (EPS-25) [43]: It consists of 25 items organized into 5 factors (suppression, avoidance, unregulated emotion, impoverished emotional experience, and signs of unprocessed emotions). The dimensions of avoidance, suppression, and unregulated emotion are elements of emotional control and describe the avoidance of stimuli that trigger emotional responses, the control of emotional states and their expression, and the lack of control over emotions reflected in behavioral or internalized responses to emotional events, respectively. The dimension of impoverished emotional experience refers to difficulties experiencing emotions, being aware of them, and identifying and distinguishing them from bodily sensations. The factor designated unprocessed emotions refers to the process by which we face difficult emotional events and adapt to them by integrating them into our lives. Each item is valued on a 10-point Likert-type scale (0 “completely disagree” to 9 “completely agree”). The range of possible responses ranges from 0 to 9, with higher scores indicating lower emotional regulatory ability. In the present study, the reliability estimated by means of Cronbach’s alpha coefficient was 0.94 (95% CI = 0.92–0.95) and ranged from 0.67 to 0.88 for each of the 5 dimensions.

The modified Symptom Severity Index for Post-traumatic Stress Disorder (PTSD) (mSSI) [44]: This is a modified and updated version of the SSI [45] used to assess the severity of PTSD symptoms based on the diagnostic criteria of the DSM-5. It consists of 21 items valued on a 4-point Likert-type scale (0 “not at all” to 3 “5 or more times a week/a lot”). It analyzes the factors re-experiencing (5 items), behavioral/cognitive avoidance (3 items), cognitive impairment and negative mood (7 items), and increased physiological reactivity (6 items). Higher scores equate to more severe symptoms. In addition to the core symptoms of PTSD, it has four items to evaluate the presence of dissociative symptoms. In the present study, the reliability estimated by means of Cronbach’s alpha coefficient was 0.93 (95% CI = 0.91–0.95).

Early Trauma Inventory Self-report (EAISR-SF) [46]: The EAISR-SF is composed of 18 items indicative of the presence or absence of different types of physical, psychological, sexual, or general trauma occurring before the age of 18. The reliability index for this scale obtained by means of Cronbach’s alpha coefficient in this study was 0.72 (95% CI = 0.65–0.79).

### 2.4. Statistical Analysis

All data analyses were performed using the Statistical Package for the Social Science (SPSS 26.0) software application (International Business Machines Corpr, Armonk, NY, USA). We performed an initial hierarchical cluster analysis followed by a two-step cluster analysis to achieve the initial objective of this research. Cluster analysis is an exploratory analysis that attempts to identify structures within the data. More specifically, it tries to identify homogeneous groups of cases if the grouping is not previously known. Being exploratory, it does not make any distinction between dependent and independent variables. The hierarchical cluster analysis was designed to establish the statistically appropriate number of clusters [47,48] and was conducted along similar lines to those described in the literature on emotional regulation and post-traumatic stress [39,42]. Hierarchical cluster analysis was performed to establish the statistically appropriate number of classes of emotional regulation, applying Ward’s agglomerative clustering method, which uses Z-scores to ensure an equal contribution to the classification and preservation of the variance of the original sub-scale. Squared Euclidean distances were used as a measure of the similarity of cases. The analysis included the five strategies of emotional regulation that make up the EPS (suppression, signs of unprocessed emotions, avoidance, unregulated emotions, and impoverished emotional experience). The two-step cluster method allows us to identify natural clusters within datasets that would otherwise not be apparent. The algorithm used by the two-step cluster method offers certain desirable characteristics that make it different from and more optimal than traditional clustering and latent class techniques, in particular, the automatic selection of the number of clusters-classes by comparing the values of a model-choice criterion across different grouping solutions, and scalability, which allows us to generate a cluster-features tree, used as a summary of the records [49]. The two-step cluster analysis again included all the variables considered in the hierarchical analysis and in the same order. All the variables were standardized. The selection of the final model used in this study was based on clinical judgement and the following statistical criteria: Schwarz Bayesian clustering criterion (Bayesian BIC reporting criterion), ratio of BIC change relative to the change for the k-1, and goodness-of-fit based on a cohesion index using the Silhouette coefficient [50,51,52]. Cluster analysis was followed by an analysis of variance (ANOVA) to identify significant differences between clusters. Finally, to contrast the main hypothesis of the study that the symptoms of post-traumatic stress would vary according to the different profiles of emotional regulation and type of IPV victimization, a covariance analysis (ANCOVA) was performed with Bonferroni post-hoc comparisons (*p* < 0.05). Factors included are the type of victimization (victims with a single report, victims with several reports by the same aggressor, and victims with several reports by different aggressors) and the emotional regulation groups found in the cluster analysis, with PTSD symptoms as a criterion variable. Since it has been established that childhood trauma can be a factor that favors emotional dysregulation [30], the decision was made to annul the effect attributable to the childhood trauma variable by including the EAISR-SF score as a covariate. The Levene test to determine the equality of variances of the groups assumes equality between them (F = 0.92, *p* = 0.49).

## 3. Results

Analyses to assess whether demographic variables (age, level of education, or time since an event was reported) were related to the symptoms of post-traumatic stress or type of victim showed statistically non-significant relationships (all *p* > 0.05). Of the participants, 29.2% were diagnosed with PTSD based on DSM-5 criteria [53]. When comparing the prevalence of PTSD in each group of victims with respect to each of the other groups, a PTSD prevalence odds ratio of 4.44 was found in the VSRDA group versus RSV (95% CI = 1.4–13.1, χ2 = 7.5, *p* = 0.006; η2 = 0.143) and 3.38 versus VSRSA (95% CI = 1.2–10.0, χ2 = 5.1, *p* = 0.02; η2 = 0.11), with no significant differences between the VSR and VSRSA groups.

**Figure 1:**
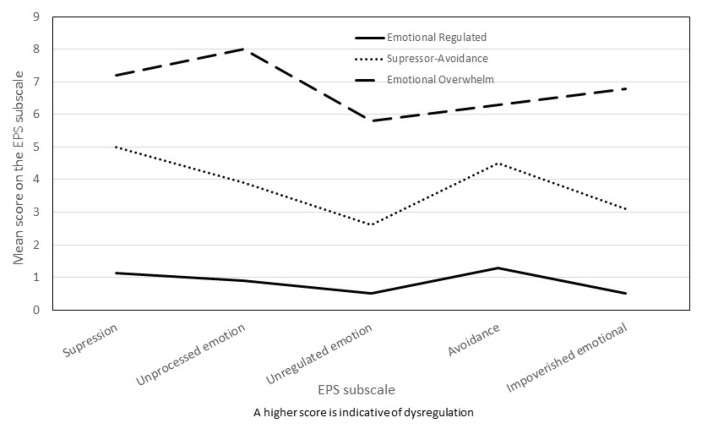
Profiles of emotional regulation in battered women.

Hierarchical cluster analysis suggested a three-cluster solution. When analyzing the fusion coefficients between cases and the dendrogram, we found that the underlying structure of the data is adequately reflected in a solution of three relatively homogeneous groups without large fusion coefficients. The automatic solution provided by two-phase cluster analysis maintained the three-group solution with a value of 0.6 for the Silhouette measure of cohesion and separation valued at the highest quality level [50]. The additional evidence for the validity of the 3 groups is adequate since it fits with the existence of an intermediate emotional regulation profile according to the literature [39,41,42] and because of the predictive validity subsequently found with respect to clinical measures [48]. Cluster 1 (*n* = 33, 27.5%) was labeled Emotional Overwhelm, Cluster 2 (*n* = 44, 36.7%) was called Avoidance/Non-Impoverished, and the third was Emotional Regulated (*n* = 43, 35.8%). The three groups do not differ in terms of demographic variables (age, level of education, or time since the report was filed, all *p* > 0.05). The mean scores for all emotional processing sub-scales included in the cluster analysis are presented in Table 1, and Figure 1 visually presents the profile of the 3 clusters extracted. The Emotional Overwhelm cluster had the highest scores on all sub-scales of the Emotional Processing Scale, the Avoidance/Non-Impoverished cluster had intermediate scores on all sub-scales, and the Emotional Regulated cluster had the lowest-value scores in all measures. The profile of the three groups maintains a common silhouette, although in the Avoidance/Non-Impoverished profile, the score on the avoidance sub-scale is close to that of the Emotional Overwhelm group, while the score on the Impoverished Emotional Experience sub-scale is close to that of the Emotional Regulated group. The effect size of the differences between the three clusters (η_p_^2^) is high for all variables, far exceeding the usual 0.14 criterion to be considered a large size [54] (see Table 1).

**Figure 2:**
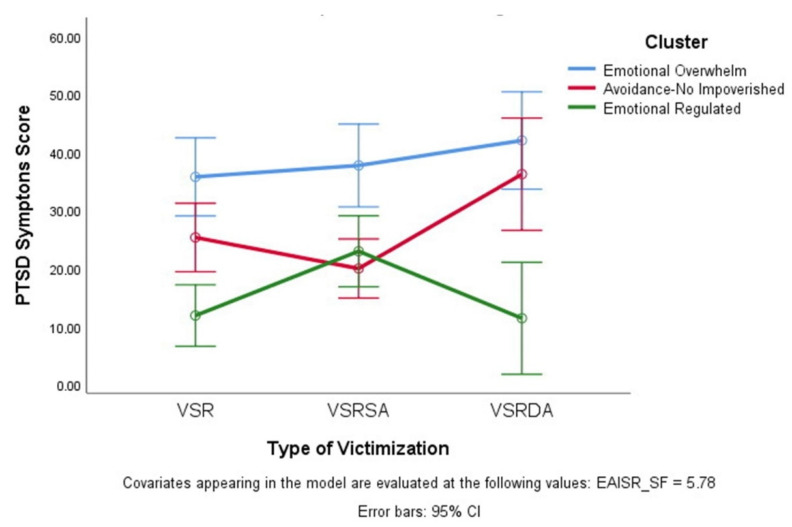
Symptoms of Post-Traumatic Stress according to type of victimization and profile of emotion regulation. VSR: victims with a single report, VSRSA: victims with several reports by the same aggressor; VSRDA: victims with several reports by different aggressors.

The results of the ANCOVA showed a significant global effect for the model (F(9.118) = 11.5, *p* < 0.001; η_p_^2^ = 0.49), a non-significant effect for type of victim, a significant effect according to the emotional regulation group (F(2.118) = 27.9, *p* < 0.001; η_p_^2^ 0.34), a significant interaction between type of victim and emotional regulation group (F(4.114) = 10.5, *p* < 0.05; η_p_^2^ = 0.09), and a significant effect for the covariable childhood trauma (F(1.114) = 7.7, *p* < 0.01; η_p_^2^ = 0.064). Post-hoc comparisons in post-traumatic stress symptoms according to the emotional regulation group showed statistically significant differences between the three possible comparisons (all *p* < 0.001). Furthermore, the interaction pattern between type of victim and the emotional regulation group showed that for VSR and VSRDA women, the Emotional Regulated group differs from the other two emotional regulation groups in post-traumatic symptoms, and that in the VSRSA group of women, the Emotional Regulated and Avoidance/Non-Impoverished groups differ from the Emotional Overwhelm group in post-traumatic symptoms (see Figure 2). Table 2 shows the mean values for PTSD symptoms in each emotional regulation group and victim type.

## 4. Discussion

Research in the field of domestic violence against women and its relationship to the subsequent development of post-traumatic stress symptoms has focused mainly on studying factors external to the victim (e.g., intensity of violence or accumulation of traumatic incidents) that favor the clinical profile, ignoring factors internal to the victim herself, which has limited research in this field [18]. Investigators might well have long avoided identifying the characteristics of the victim that may increase the risk of re-abuse in an attempt to avoid victim-blaming when the focus shifts away from the aggressors [35]. Although this is a legitimate concern, it is important to collect information on the vulnerability of victims as it could guide patterns of action or treatment aimed at women’s empowerment and prevent revictimization by favoring the recovery of people subjected to intimate partner violence [35,55].

Along these lines, the present study sought to relate a multidimensional measure of emotional regulation, expressed as profiles, to the severity of PTSD symptoms in a sample of women victims of IPV.

We observed a high presence of post-traumatic symptoms in women victims of IPV. Almost one third of the sample fulfilled the diagnostic criteria for PTSD, consistent with other studies that indicate that the prevalence of this disorder within this population is almost eight times higher than in the general population and nearly five times that of people exposed to other traumatic incidents [13,56].

The results showed the presence of three different profiles of emotional regulation, called Emotional Overwhelm, Avoidance/Non-Impoverished, and Emotional Regulated, respectively. The Emotional Overwhelm profile is characterized by high scores in all five strategies of emotional regulation, which is indicative of generalized emotional dysregulation. The Emotional Regulated profile has low scores for dysregulation in the five strategies, which is indicative of emotional adjustment. The Avoidance/Non-Impoverished profile has intermediate scores on all sub-scales. When the silhouette is compared between the three profiles, a certain similarity is observed between them that is broken in the Avoidance/Non-Impoverished profile as it approaches the Emotional Overwhelm group in the avoidance score and moves markedly away from it in signs of emotional impoverishment (see Figure 1). Analyzing how PTSD diagnoses are divided between the 3 profiles shows that in the group of women with a high level of dysregulation, there were 64% PTSD diagnoses, 27% in the Avoidance/Non-Impoverished group, and only 4% in the Emotional Regulated group. In addition, the overall severity of PTSD was significantly different in the three emotional regulation profiles, highest in the Emotional Overwhelm group. This offers not only a new empirical perspective on the relationship between emotional regulation and PTSD in IPV victims [39], but also additional evidence for validity in cluster selection. The choice of three clusters allows for more refined discrimination as to how women subjected to IPV regulate emotionally [48]. Our findings are consistent with the global literature on emotional regulation and intensity of PTSD symptoms that suggests a general lack of emotional regulation in people exposed to various traumatic events and which, in this study, extends to women who have experienced IPV [28]. This appears to be confirmed by the high score in all dimensions of emotional regulation found in the profile with more intense PTSD symptoms and in most people with a PTSD diagnosis. This low capacity to regulate emotions may be reflecting a general deficit in the flexibility of emotional regulation [29,57]. In addition, the results suggest that not only is there general dysregulation, but also that women victims of IPV with greater PTSD symptomatology more frequently use any of the five evaluated emotional regulation strategies that are maladaptive to emotional homeostasis. In this sense, the Emotional Overwhelm profile, predictive of greater PTSD symptomatology, is characterized by a high score in suppression, signs of unprocessed emotions, unregulated emotions, avoidance, and emotional impoverishment. This profile is not surprising in light of the previous literature, as the strategies of suppression, avoidance, and rumination have been repeatedly identified as the maladaptive elements of emotional dysregulation contributing to PTSD [28,41,58]. The absence of emotional processing that characterizes Emotional Overwhelm and Avoidance/Non-Impoverished profiles favors the characteristic rumination present in PTSD psychopathology and helps to configure the symptoms of PTSD Cluster B [59]. Finally, the emotional impoverishment that also characterizes the Emotional Overwhelm profile is probably a reflection of difficulties in the clarity and emotional awareness of the person exposed to traumatic events who develops PTSD [26,29,60] and relates to both early traumatic events [61] and revictimization [35,36]. Emotional distancing can negatively affect the ability to detect risk and thus make it easier to become a potential target for aggressors [62,63].

## 5. Conclusions

There is ample evidence that the accumulation of IPV traumatic events generates a greater impact than single victimizations, with greater intensity in the symptoms of post-traumatic stress and greater difficulty recovering [35,64]. Since it has been argued that revictimization has been associated with weaker emotional regulation strategies [34], this study chose to contrast the existence of specific emotional dysregulation in revictimized women. Although it was found that belonging to the different regulation groups is associated with different degrees of PTSD symptoms, we did not verify the existence of differences in PTSD symptoms between the different types of victims. However, there was an interaction effect observed between emotional regulation and the type of victim. It is particularly interesting to observe the greater PTSD symptomatology among women victims by different aggressors in the two groups with greater emotional dysregulation and fewer symptoms in the group of women with good emotional regulation compared to the other two types of victims (see Figure 2). In this regard, we suggest that being the victim of several episodes of intimate partner violence at the hands of different aggressors should be understood as a form of greater severity due to the greater associated psychopathology and poorer emotional regulation compared to women victims of a single episode of violence or women who are revictimized by the same aggressor [64]. The idea that repeated victimization or poly-victimization (accumulation of different types of abuse) are the only factors related to problems of regulating emotions is not confirmed here [29,65]. Even among women who have experienced single episodes of IPV, there may also be emotional dysregulation, as shown by the fact that 28% of women victims of a single episode of violence in this study belong to the Emotional Overwhelm profile. Rather, our results support the notion described by Lilly, London, and Bridgett [30] of the existence of a particular difficulty in regulating emotions over PTSD symptoms occurring in adulthood and an indirect effect of child abuse on PTSD symptoms that may occur through emotional dysregulation at earlier ages. Our results show how, by introducing child abuse as a covariate, it is seen to be significantly and modestly related to current post-traumatic stress (η2 = 0.064), although the model remains significant with an important effect for emotional regulation on post-traumatic stress independent of child abuse (η2 = 0.34); therefore, dysregulation specific to adulthood should be deduced.

This work potentially has a significant number of limitations. Firstly, the findings might reflect a lack of specificity within the emotional regulation measure used because it does not specifically assess the nature and intensity of the emotions that women are regulating, i.e., it does not specify the context around which they regulate emotions [42]. Secondly, the generalization of the results obtained is limited to abused women who have filed a police complaint against their partner and have agreed to participate in the study. Thirdly, the cross-sectional design does not allow for the examination of causal relationships, so we cannot conclude whether dysregulation is a cause or consequence of clinical symptoms. Fourthly, the typologies of emotional regulation have been identified by means of cluster analysis rather than latent class analysis, which is understood to be superior in terms of reliability and the ability to examine fit indices. However, the relatively small sample size, the idea that in applied research cluster analysis can better guide the best model that should be interpreted [66], and the fact that similar results are found when applying both procedures to clinical samples [67] lessens the importance of the limitation caused by using a cluster analysis procedure. Finally, the study only examined certain forms of emotional regulation, not all of them, so it would be interesting to carry out research that would take into account a greater number of dimensions.

It is important to emphasize that the research is limited to conducting an emotional and behavioral analysis of emotional regulation in women victims of IPV who suffer from PTSD. A global understanding of this phenomenon implies a bio-psycho-socio-ecological model that contemplates the life course perspective to determine risk and resilience [68]. The biological footprint of environmental adversity may be key to understanding the processes of loss of health, as it encompasses both the damage induced as well as the compensatory reactions of the organism. IPV can deregulate and recalibrate environmentally sensitive physiological (i.e., central nervous, endocrine, and immune) systems, putting women who have experienced an IPV incident at risk for multiple health problems. It has also been noted that the heterogeneity of PTSD indicates the presence of multiple neurobiological mechanisms underlying the etiology and maintenance of PTSD [69]. Translational research in recent decades is revealing different potential pathways for the identification of PTSD biomarkers. Some of them would be monoaminergic transmitter systems, the hypothalamic–pituitary–adrenal (HPA) axis, metabolic hormonal pathways, inflammatory mechanisms, psychophysiological reactivity, and neural circuits. It is likely that these biomarkers will be identified in the future from multidimensional models that include comprehensive descriptions of molecular, neurobiological, behavioral, and clinical phenotypes [69,70]. A good example of this is the PTSD Biomarker Database, a database that provides a general view of physiological markers being studied as putative biomarkers in the current post-traumatic stress disorder (PTSD) literature that allows researchers to compare findings quickly [71]. This database currently contains over 900 biomarkers. Currently, there is a complementary effort to identify objective blood gene expression biomarkers for psychological stress in the area of personalized medicine [70].

The findings of this paper have substantial clinical implications. This research aims to complement the standard PTSD intervention in women victims of IPV through emotion-centered interventions. Cognitive behavioral therapy is not always effective when there is intense affective dysregulation [72], so victims can benefit from acquiring skills to regulate affect [64]. In addition, women who have been revictimized on several occasions, with a history of accumulated victimization, might respond better to therapy if problems regulating affect are addressed directly [62]. From a preventive perspective, it should be assessed whether intervention programs for first IPV episodes favor women’s empowerment and prevent revictimization.

Research has not yet established the value of jointly using emotional regulation strategies in relation to traumatic events, especially in the area of partner violence. We believe that this study contributes to this purpose through a person-centered approach by examining a wide range of emotional regulation strategies. The results show the existence of different profiles of emotional regulation in women exposed to intimate partner violence that translate into different intensities of post-traumatic stress, a difficulty that increases among women who have been the victims of repeated IPV incidents at the hands of several partners.

## Figures and Tables

**Table 1:** Distribution of the three subtypes on the Emotional Processing Scale (EPS) sub-scales included in the two-step cluster analysis.

EPS Sub-Scales	Emotional Overwhelm (*n* = 33)	Avoidance/ Non-Impoverished (*n* = 44)	Emotional Regulated (*n* = 43)	Total Sample (*n* = 120)	F(2, 120)	η2
Suppression	7.2 (1.3)	5.0 (1.6)	1.1 (1.2)	4.2 (2.8)	189.0 **	0.76
Signs of Unprocessed Emotion	8.0 (3.4)	3.9 (1.7)	0.9 (1.2)	4.0 (3.5)	94.6 **	0.62
Unregulated Emotion	5.8 (1.7)	2.6 (1.6)	0.5 (0.6)	2.7 (2.5)	141.1 **	0.70
Avoidance	6.3 (1.8)	4.5 (1.7)	1.3 (1.3)	2.7 (2.5)	90.3 **	0.61
Impoverished Emotional Experience	6.8 (3.7)	3.1 (1.5)	0.5 (0.7)	3.9 (2.6)	73.0 **	0.56
EPS Total Score	6.7 (3.7)	2.7 (1.7)	0.4 (0.6)	3.0 (2.6)	344.5 **	0.86

Note. Means are displayed along with standard deviations (SD); ** *p* < 0.001. EPS Total Score = Global score of the Emotional Processing Scale.

**Table 2:** Mean of PTSD symptomatology for each profile of emotional regulation.

	VSR (*n* = 51)	VSRSA (*n* = 49)	VSRDA (*n* = 20)	Total Sample (*n* = 120)
Emotional Regulated (*n* = 43)	10.1 (8.3)	22.5 (12.8)	10.7 (5.2)	14.8 (11.4)
Avoidance/Non-Impoverished (*n* = 44)	24.9 (10.5)	21.1 (13.7)	37.5 (16.1)	24.7 (13.8)
Emotional Overwhelm (*n* = 33)	38.7 (12.6)	36.8 (14.6)	43.4 (13.9)	39.2 (13.4)
Total Sample	22.6 (15.4)	25.1 (14.8)	31.8 (18.8)	25.1 (16.0)

Means are displayed along with standard deviations (SD). VSR: victims with a single report, VSRSA: victims with several reports by the same aggressor, VSRDA: victims with several reports by different aggressors.

## Data Availability

The data presented in this study are available on request from the corresponding author. The data are not publicly available due to ethical standards and legal requirements.
